# Exploiting Joint Base Station Equipped Multiple Antenna and Full-Duplex D2D Users in Power Domain Division Based Multiple Access Networks

**DOI:** 10.3390/s19112475

**Published:** 2019-05-30

**Authors:** Dinh-Thuan Do, Minh-Sang Van Nguyen, Thi-Anh Hoang, Byung Moo Lee

**Affiliations:** 1 Wireless Communications Research Group, Faculty of Electrical and Electronics Engineering, Ton Duc Thang University, Ho Chi Minh City 700000, Vietnam; 2 Faculty of Electronics Technology, Industrial University of Ho Chi Minh City (IUH), Ho Chi Minh City 700000, Vietnam; nguyenvanminhsang@iuh.edu.vn (M.-S.V.N.); hoangthianh@iuh.edu.vn (T.-A.H.); 3 School of Intelligent Mechatronics Engineering, Sejong University, Seoul 05006, Korea

**Keywords:** full-duplex, multiple input single output, power domain division based multiple access

## Abstract

In this paper, we investigate power domain division-based multiple access (PDMA) to support the base stations (BS) equipped with multiple antennas to serve mobile users. Such a system deploys multiple input single output (MISO)-based wireless transmission and a full-duplex (FD) scheme. Furthermore, such MISO PDMA system consists of BS employing transmit antenna selection to reduce complexity in signal processing at the receivers. We distinguish two kinds of mobile users, device-to-device (D2D) users and traditional users. In such MISO PDMA, there exists a trade-off between outage performance of each PDMA user and power allocation factors. Since the implementation of the FD scheme at PDMA users, bandwidth efficiency will be enhanced despite the existence of self-interference related to such FD. In particular, exact expressions of outage probability are derived to exhibit system performance with respect to D2D users. Finally, valuable results from the simulated parameters together with the analytical results show that MISO PDMA can improve its performance by increasing the number of transmit antennas at the BS.

## 1. Introduction

To adapt fast development in wireless techniques, many protocols and topologies have been introduced. One of these schemes, relaying networks has recently attracted the research community due to extended coverage and improved reliability [1,2,3,4]. Full-duplex is considered a scheme that exhibits higher bandwidth efficiency and such schemes are proposed in wireless powered relaying networks as in [2,3]. However, multiple users need to be served to access the core network, and it requires the base station in a cellular network that can transmit a mixture of signals to them. To address this shortcoming, PDMA or non-orthogonal multiple access (NOMA) has been recently introduced to improve the spectral efficiency and to provide fairness in resource allocation. These advantages can be performed by multiplexing multiple users on the same time/frequency resource [5,6,7,8,9,10,11,12]. In particular, NOMA employs the power domain to serve multiple access and harnesses interference via superposition coding at the transmitter, while successive interference cancellation (SIC) is required at the receiver. In [6], the authors investigated maximal performance of single-input single output (SISO) single-carrier (SC) NOMA systems in terms of system throughput and they explored the optimal power allocation design. The higher spectral efficiency can be achieved in SISO NOMA compared to conventional SISO OMA [6]. The suboptimal precoding design is presented for minimization of the transmit power in multiple-input single-output (MISO) SC-NOMA systems [7]. In addition, zero-forcing downlink (DL) beamforming was analyzed in MISO SC-NOMA systems [8].

Furthermore, it is very expensive to deploy a MISO system since both space-time codes and transmit beamforming require multiple RF chains [13]. Fortunately, the antenna selection scheme is proposed to overcome such disadvantage, providing a good trade-off between cost, complexity, and performance [13]. Antenna selection can be implemented at both ends, and transmit antenna selection with maximal-ratio combining (TAS/MRC) is presented in [14]. This scheme can be mainly described as follows: by using CSI feedback, the best transmit antenna out of all transmit candidates to maximize the post processing signal to noise ratio (SNR) at the MRC output of receive antennas, is selected to transmit data for the corresponding user. These analytical discussions motivate us to explore improved performance of MISO PDMA.

With distant transmission, relaying schemes are required but in close distance transmission, and so new protocols need to be exhibited. Recently, in project 3GPP for long term evolution (LTE), device to device (D2D) communication has been introduced. As one of the effective technologies of the forthcoming 5th generation (5G) cellular standard, D2D is explored as in [15]. By using new paradigm, i.e., without or limited controlling and signaling information from the base station (BS), two users can communicate in instant and direct ways with each other (when in proximity) in context of D2D scenario [16,17,18,19,20]. Furthermore, potential application in disaster-affected areas needs fast connections and D2D can be adopted in such case. In particular, the local connectivity is provided to devices even in a case of damage to the network infrastructure. D2D can be employed in several other emerging applications. For example, vehicular-to-vehicular (V to V) communication, vehicular-to-infrastructure (V to I) communication are introduced with applications of D2D communication to exhibit proximity based add-on services and multi-party gaming or public safety applications are studied as well [21,22]. It can be exhibited commercial D2D communication to improve the throughput, spectrum utilization, and energy efficiency of the cellular network. Other challenges are raised such as interference management security. To meet the capacity requirements of the 5G cellular system, a project was deployed and it is known as METIS (mobile and wireless communications enablers for the twenty-twenty information society). The METIS has recently been funded by the European Union [23].

Regarding exploiting advantages of D2D into NOMA, the authors in [24] investigated a model of the integration of a downlink NOMA system with D2D communications. D2D reported in [25] with resource allocation scheme is promising approach. They further derived expressions of the outage probability that both users obtain higher rates in NOMA under a fixed power control strategy. In addition, the uplink multi-carrier is considered in NOMA with support of D2D underlaid cellular networks [26]. More specifically, an iterative algorithm applying Karush-Kuhn-Tucker conditions is proposed to solve the power allocation problem in D2D NOMA [26]. The authors in [27] studied the device-to-device (D2D) assisted and NOMA-based mobile edge computing (MEC) system by deploying D2D communication for enabling user collaboration and reducing the edge server’s load.

In this paper, we consider a D2D transmission existing in a downlink PDMA system. The selected antenna at one BS communicating has two D2D far receivers with the aid of D2D implementation. Different from existing works on D2D PDMA [26,27], where the end-user operating half-duplex, we assume that the D2D users operate in the FD mode and investigate outage performance taking into account both the downlink and D2D links. The key contributions of this study are summarized as follows:

- In the presence of a downlink under support of multiple antenna based BS, two D2D users exhibit different outage performance. We individually investigated the performance of each end-user in such a MISO NOMA system. Compared to most existing cooperative PDMA schemes, FD scheme is enabled at the end-user. To look how good performance two far users have, two far D2D users with different QoS requirements can be paired with each other and get benefit from D2D transmission.
- Different from [25], transmit selection and full-duplex are joint investigated in this study. Most important is that we provide simulation results of integration of a D2D connection to a downlink two-user PDMA system.
- We provide simulation results showing that, under the fixed power allocation strategies, D2D users achieve outage behavior in the NOMA scheme. The results also indicate the probability that both D2D users obtain improved outage performance in MISO PDMA depends on the power level of the BS and the required target rates.
- For system performance evaluation, the closed-form expressions for the outage probabilities are derived for both two D2D users. To highlight the impact of the system parameters on the outage performance, outage probabilities achieved at both two D2D users are simulated to verify derived expressions.

**Notation:** The cumulative distribution function of a real-valued random variable *X* is denoted by $F_{X}\left(.\right)$, $f_{X}\left(.\right)$ stands for probability density functions, while Pr(.) symbolizes outage probability.

## 2. System Model

**Figure 1:**
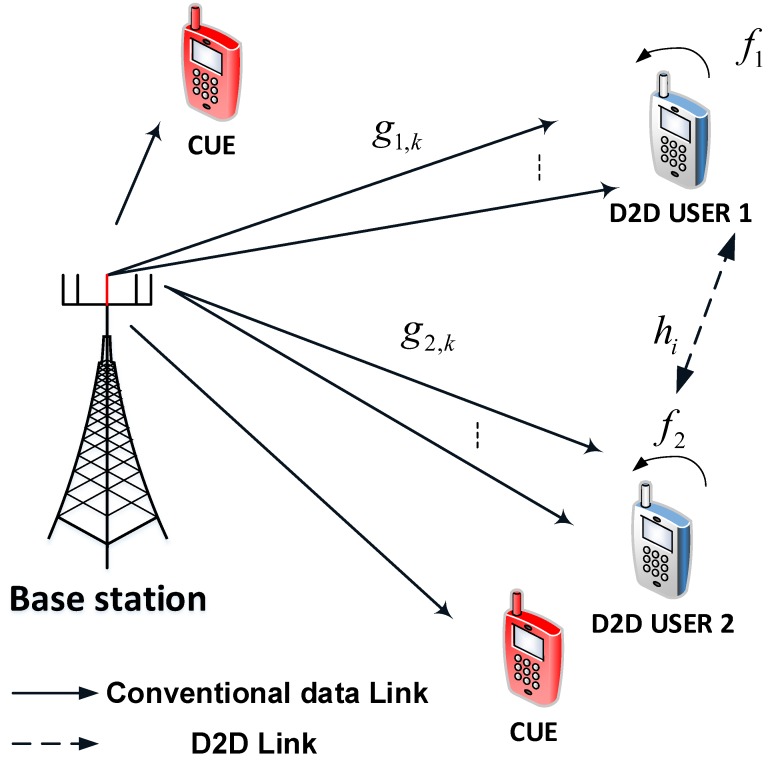
System model of FD aware D2D transmission mode in Power Domain based Multiple Access with Multiple Antenna at the BS.

We consider a downlink MISO aided PDMA network as shown in Figure 1, in which the base station (BS) is equipped with multiple antennas to serve two PDMA users. There are conventional cellular users (CUE) in this model, such CUE devices are able to receive signal under coverage of this BS, but this paper focuses on more complex operations of D2D users. It is assumed that interference from CUE to D2D users is smaller than self-interference at each D2D user. In this case, two PDMA users ($D_{1},D_{2}$) operate in full-duplex (FD) mode and they can communicate directly without helping of the BS as underlay topology. Two PDMA users are able to communicate directly on channel $h_{i},i=1,2$. It is noted that $g_{i,k}$ denotes the channel gain between the BS and user $D_{i},i=1,2$, the BS has k,(k=1,2,…K) antennas. Further, in this scenario PDMA users are double-antenna devices and operate in a FD mode, except for the BS equipped multiple antenna. The direct links between the source node and the users are assumed available, which is common in the scenarios where two PDMA users acquire device to device transmission in reliable coverage of such BS. We assume that all users are clustered very close such that a homogeneous network topology is considered in our paper. The channels associated with each link exhibit the Rayleigh fading and additive white Gaussian noise (AWGN).

In first phase, the BS sends signal $x=\sqrt{a_{1}P_{S}}x_{1}+\sqrt{a_{2}P_{S}}x_{2}$ to $D_{1}$ and $D_{2}$ according to direct transmissions. Here, $P_{S}$ is the transmitted power of the BS, $x_{1}\left(x_{2}\right)$ is the signal of $D_{1}\left(D_{2}\right)$ , and $a_{1}$ , $a_{2}$ is the power allocation coefficient with $a_{1}+a_{2}=1,a_{1}>a_{2}$.

### 2.1. Calculation of Signal to Noise Ratio (SNR)

The received signal at $D_{i}$ is given by

(1) $$y_{Di}^{FD-PDMA}=g_{i,k}\left(\sqrt{a_{1}P_{S}}x_{1}+\sqrt{a_{2}P_{S}}x_{2}\right)+f_{i}\sqrt{\varpi P_{Di}}x_{FDi}+w_{i},$$

where ϖ=1 denotes user 1 working in FD, $P_{Di}$ is transmit power of $D_{i},i=1,2$ and $w_{i}$ is the additive white Gaussian noise with zero mean and variance $N_{0}$. We call $x_{FDi}$ a signal related to self-interference at user *i*, and $f_{i}$ is the self-interference channel and follows $f_{i}∼CN\left(0,\lambda_{f_{i}}\right)$.

Then, the received signal-to-interference-plus-noise ratio (SINR) at user 1 $D_{1}$ becomes

(2) $$y_{SD1,k}^{FD-PDMA}=\frac{a_{1}\rho {\left|g_{1,k}\right|}^{2}}{a_{2}\rho {\left|g_{1,k}\right|}^{2}+\varpi \rho {\left|f_{1}\right|}^{2}+1},$$

where ρ=PSPSN0N0 is the transmit signal-to-noise ratio (SNR) which was measured at the BS.

In this scenario, $D_{2}$ is so-called as SIC user, i.e., SIC is required to eliminate interference from signal of $D_{1}$. Firstly, the received SINR at user 2 to detect user1’s message $x_{1}$ is given by

(3) $$\gamma_{SD1\leftarrow 2,k}^{FD-PDMA}=\frac{a_{1}\rho {\left|g_{2,k}\right|}^{2}}{a_{2}\rho {\left|g_{2,k}\right|}^{2}+\varpi \rho {\left|f_{2}\right|}^{2}+1}.$$

Then SIC is activated to eliminate interference from $D_{1}$, the received SINRs at the user 2 $D_{2}$ is calculated to decode its own signal as

(4) $$\gamma_{SD2,k}^{FD-PDMA}=\frac{a_{2}\rho {\left|g_{2,k}\right|}^{2}}{\varpi \rho {\left|f_{2}\right|}^{2}+1}.$$

### 2.2. D2D Transmission

In this phase, the cooperation signal is transmitted from the user with a stronger channel gain to the user with a weaker gain. The cooperation signal can help user 1 to decode its data, or user 2 to perform SIC better. The cooperation signal received by user 1 $D_{1}$ is given by

(5) $$z_{Di}^{PDMA}=h_{i}\sqrt{P_{S}}s+f_{i}\sqrt{\varpi P_{Di}}x_{FDi}+n_{i},$$

where $h_{1}$ is a Rayleigh fading channel coefficient from user 1 to user 2 and vice versa. As mentioned in the channel information exchange phase, when ${\left|g_{1,k}\right|}^{2}>{\left|g_{2,k}\right|}^{2}$ , only $z_{D2}^{PDMA}$ exists, and when ${\left|g_{1,k}\right|}^{2}<{\left|g_{2,k}\right|}^{2}$ , only $z_{D1}^{PDMA}$ is transmitted from $D_{2}$.

Generally, the received SINR at user *i* is given by

(6) $$\chi_{Di}^{PDMA}=\frac{\rho {\left|h_{i}\right|}^{2}}{\varpi \rho {\left|f_{i}\right|}^{2}+1}.$$

The SINR for decoding $x_{1}$ is given by

(7) $$\chi =\left\{\begin{array}{c} \min\left\{\max\left\{\gamma_{SD1,k}^{PDMA},\chi_{D1}^{PDMA}\right\},\gamma_{SD1\leftarrow 2,k}^{PDMA}\right\},\mathrm{if}{\left|g_{1,k}\right|}^{2}<{\left|g_{2,k}\right|}^{2}\\ \min\left\{\gamma_{SD1,k}^{PDMA},\max\left\{\gamma_{SD1\leftarrow 2,k}^{PDMA},\chi_{D2}^{PDMA}\right\}\right\},\mathrm{otherwise} \end{array}\right..$$

The antenna index can be selected to strengthen the BS to serve user *i* link as follows:

(8) $$k^{*}=\arg\underset{k=1,\ldots ,K}{\underset{︸}{max}}\left({\left|g_{i,k}\right|}^{2}\right).$$

In this case, CDF and PDF related selected channel are given respectively by

(9) $$F_{{\left|g_{i,k^{*}}\right|}^{2}}\left(x\right)=1-\sum_{k=1}^{K}\left(\begin{array}{c} K\\ k \end{array}\right){\left(-1\right)}^{k-1}\exp\left(-\frac{kx}{\lambda_{g}}\right),$$

and

(10) $$f_{{\left|g_{i,k^{*}}\right|}^{2}}\left(x\right)=\sum_{k=1}^{K}\left(\begin{array}{c} K\\ k \end{array}\right){\left(-1\right)}^{k-1}\frac{k}{\lambda_{g}}\exp\left(-\frac{kx}{\lambda_{g}}\right).$$

Here, $\lambda_{u}$ is the channel gain of *u*.

## 3. Outage Probability Performance Analysis

When the targeted data rates, $R_{1}$ and $R_{2}$ are determined by the users’ QoS requirements for user $D_{1},D_{2}$. In fact, the outage probability is an important performance criterion which needs to be investigated. If the outage event occurs at the non-SIC user, the SIC user does not use the D2D signal, and the outage of the SIC user does not allow D2D transmission from the SIC user to the non-SIC user.

### 3.1. Outage Probability of D2D User 1

Considering outage probability of $D_{1}$: According to PDMA protocol, the complementary events of outage at $D_{1}$ can be explained as: $D_{1}$ can detect $x_{2}$ as well as its own message $x_{1}$. From the above description, the outage probability of $D_{1}$ is expressed as

(11) $$\begin{array}{c} OP_{D1-bi}=\underset{B_{1}}{\underset{︸}{\mathrm{Pr}\left(\gamma_{SD1,k*}^{PDMA}<\varepsilon_{1},\gamma_{SD1\leftarrow 2,k*}^{PDMA}<\varepsilon_{1}\right)}}\\ +\underset{B_{2}}{\underset{︸}{\mathrm{Pr}\left(\max\left\{\gamma_{SD1,k*}^{PDMA},\chi_{D1}^{PDMA}\right\}<\varepsilon_{1},\gamma_{SD1\leftarrow 2,k*}^{PDMA}>\varepsilon_{1}\right)}}, \end{array}$$

**Proposition** **1.** *The closed-form expression of outage probability at D1 is given by*

(12) $$\begin{array}{c} OP_{D1-bi}=\left(1-\sum_{k=1}^{K}\left(\begin{array}{c} K\\ k \end{array}\right){\left(-1\right)}^{k-1}\vartheta_{1}\right)\left(1-\sum_{k=1}^{K}\left(\begin{array}{c} K\\ k \end{array}\right){\left(-1\right)}^{k-1}\vartheta_{2}\right)\\ +\left(1-\sum_{k=1}^{K}\left(\begin{array}{c} K\\ k \end{array}\right){\left(-1\right)}^{k-1}\vartheta_{1}\right)\\ \times \left(1-\frac{\rho \lambda_{h_{1}}}{\rho \lambda_{h_{1}}+\varepsilon_{1}\varpi \rho \lambda_{f_{1}}}\exp\left(-\frac{\varepsilon_{1}}{\rho \lambda_{h_{1}}}\right)\right)\sum_{k=1}^{K}\left(\begin{array}{c} K\\ k \end{array}\right){\left(-1\right)}^{k-1}\vartheta_{2} \end{array}$$

*where*$\varepsilon_{1}=2^{2R_{1}}$, $R_{1}$*is target rate for signal*$x_{1}$*.*

**Proof.**  See Appendix A. □

### 3.2. Outage Probability of D2D User 2

The outage events of $D_{2}$ can be explained as below. The first is that $D_{1}$ cannot detect $x_{2}$. The second is that $D_{2}$ cannot detect its own message $x_{2}$ on the conditions that $D_{1}$ can detect $x_{2}$ successfully. Based on these, the outage probability of $D_{2}$ is expressed as

(13) $$\begin{array}{c} OP_{D2-bi}=\underset{\Psi_{1}}{\underset{︸}{\mathrm{Pr}\left(\begin{array}{c} \left(\gamma_{SD2,k*}^{PDMA}<\varepsilon_{2}\cup \gamma_{SD1\leftarrow 2,k*}^{PDMA}<\varepsilon_{1}\right),\\ \gamma_{SD1,k*}^{PDMA}<\varepsilon_{2} \end{array}\right)}}\\ +\underset{\Psi_{2}}{\underset{︸}{\mathrm{Pr}\left(\begin{array}{c} \gamma_{SD2,k*}^{PDMA}<\varepsilon_{2}\cup \max\left\{\gamma_{SD1\leftarrow 2,k*}^{PDMA},\chi_{D2}^{PDMA}\right\}<\varepsilon_{1},\\ \gamma_{SD1,k*}^{PDMA}>\varepsilon_{1} \end{array}\right)}}, \end{array}$$

where $\varepsilon_{2}=2^{2R_{2}}$, $R_{2}$ is denoted as target rate for signal $x_{2}$, and with the help of (5) and (7), terms $\Psi_{1}$ and $\Psi_{2}$ can be calculated, the first being

(14) $$\begin{array}{c} \Psi_{1}=\mathrm{Pr}\left(\left(\gamma_{SD2,k*}^{PDMA}<\varepsilon_{2}\cup \gamma_{SD1\leftarrow 2,k*}^{PDMA}<\varepsilon_{1}\right),\gamma_{SD1,k*}^{PDMA}<\varepsilon_{2}\right)\\ =\underset{D_{11}}{\underset{︸}{\left(1-\mathrm{Pr}\left(\gamma_{SD2,k*}^{PDMA}\geq \varepsilon_{2},\gamma_{SD1\leftarrow 2,k*}^{PDMA}\geq \varepsilon_{1}\right)\right)}}\underset{D_{12}}{\underset{︸}{\left(1-\mathrm{Pr}\left(\gamma_{SD1,k*}^{PDMA}\geq \varepsilon_{2}\right)\right)}}. \end{array}$$

Therefore, $D_{11}$ can be expressed as

(15) $$\begin{array}{c} D_{11}=\mathrm{Pr}\left({\left|g_{2,k}\right|}^{2}\geq \frac{\varepsilon_{2}\varpi \rho {\left|f_{2}\right|}^{2}+\varepsilon_{2}}{a_{2}\rho },{\left|g_{2,k}\right|}^{2}\geq \frac{\varepsilon_{1}\varpi \rho {\left|f_{2}\right|}^{2}+\varepsilon_{1}}{a_{1}\rho -\varepsilon_{1}a_{2}\rho }\right)\\ =\mathrm{Pr}\left({\left|g_{2,k}\right|}^{2}\geq \frac{\varepsilon_{2}\left(\varpi \rho {\left|f_{2}\right|}^{2}+1\right)}{a_{2}\rho },{\left|g_{2,k}\right|}^{2}\geq \frac{\varepsilon_{1}\left(\varpi \rho {\left|f_{2}\right|}^{2}+1\right)}{a_{1}\rho -\varepsilon_{1}a_{2}\rho }\right)\\ =\mathrm{Pr}\left({\left|g_{2,k}\right|}^{2}\geq \left(\varpi \rho {\left|f_{2}\right|}^{2}+1\right)\max\left(\frac{\varepsilon_{2}}{a_{2}\rho },\frac{\varepsilon_{1}}{a_{1}\rho -\varepsilon_{1}a_{2}\rho }\right)\right), \end{array}$$

where $\theta =\max\left(\frac{\varepsilon_{2}}{a_{2}\rho },\frac{\varepsilon_{1}}{a_{1}\rho -\varepsilon_{1}a_{2}\rho }\right)$.

It worth noting that, we can achieve important computations as below:

(16) $$\begin{array}{c} D_{11}=\mathrm{Pr}\left({\left|g_{2,k}\right|}^{2}\geq \left(\varpi \rho {\left|f_{2}\right|}^{2}+1\right)\theta \right)\\ =\frac{1}{\lambda_{f_{2}}}\int_{0}^{\infty }\sum_{k=1}^{K}\left(\begin{array}{c} K\\ k \end{array}\right){\left(-1\right)}^{k-1}\exp\left(-\frac{\left(\varpi \rho y+1\right)\theta k}{\lambda_{2}}-\frac{y}{\lambda_{f_{2}}}\right)dy\\ =\sum_{k=1}^{K}\left(\begin{array}{c} K\\ k \end{array}\right){\left(-1\right)}^{k-1}\frac{\lambda_{2}}{\theta k\varpi \rho \lambda_{f_{2}}+\lambda_{2}}\exp\left(-\frac{\theta k}{\lambda_{2}}\right). \end{array}$$

Similarly, $D_{12}$ can be expressed as

(17) $$\begin{array}{c} D_{12}=1-\mathrm{Pr}\left(\gamma_{SD1,k*}^{PDMA}\geq \varepsilon_{2}\right)\\ =1-\mathrm{Pr}\left({\left|g_{1,k}\right|}^{2}\geq \frac{\varepsilon_{2}\varpi \rho {\left|f_{1}\right|}^{2}+\varepsilon_{2}}{a_{1}\rho -\varepsilon_{2}a_{2}\rho }\right)\\ =1-\frac{1}{\lambda_{f_{1}}}\int_{0}^{\infty }\sum_{k=1}^{K}\left(\begin{array}{c} K\\ k \end{array}\right){\left(-1\right)}^{k-1}\exp\left(-\frac{\left(\varepsilon_{2}\varpi \rho x+\varepsilon_{2}\right)k}{\left(a_{1}\rho -\varepsilon_{2}a_{2}\rho \right)\lambda_{1}}-\frac{x}{\lambda_{f_{1}}}\right)dx\\ =1-\sum_{k=1}^{K}\left(\begin{array}{c} K\\ k \end{array}\right){\left(-1\right)}^{k-1}\upsilon_{1}, \end{array}$$

where $\upsilon_{1}=\frac{\left(a_{1}\rho -\varepsilon_{2}a_{2}\rho \right)\lambda_{1}}{\left(a_{1}\rho -\varepsilon_{2}a_{2}\rho \right)\lambda_{1}+\varepsilon_{2}\varpi \rho k\lambda_{f_{1}}}\exp\left(-\frac{\varepsilon_{2}k}{\left(a_{1}\rho -\varepsilon_{2}a_{2}\rho \right)\lambda_{1}}\right)$.

From (16) and (17), we find the expression $\Psi_{1}$

(18) $$\begin{array}{c} \Psi_{1}=\left(1-\sum_{k=1}^{K}\left(\begin{array}{c} K\\ k \end{array}\right){\left(-1\right)}^{k-1}\frac{\lambda_{2}}{\theta k\varpi \rho \lambda_{f_{2}}+\lambda_{2}}\exp\left(-\frac{\theta k}{\lambda_{2}}\right)\right)\\ \times \left(1-\sum_{k=1}^{K}\left(\begin{array}{c} K\\ k \end{array}\right){\left(-1\right)}^{k-1}\upsilon_{1}\right). \end{array}$$

Next, $\Psi_{2}$ can be calculated by

(19) $$\begin{array}{c} \Psi_{2}=\mathrm{Pr}\left(\gamma_{SD2,k*}^{PDMA}<\varepsilon_{2}\cup \max\left\{\gamma_{SD1\leftarrow 2,k*}^{PDMA},\chi_{D2}^{PDMA}\right\}<\varepsilon_{1},\gamma_{SD1,k*}^{PDMA}>\varepsilon_{1}\right)\\ =\left[\mathrm{Pr}\left(\gamma_{SD2,k*}^{PDMA}<\varepsilon_{2}\right)+\underset{D_{21}}{\underset{︸}{\mathrm{Pr}\left(\max\left\{\gamma_{SD1\leftarrow 2,k*}^{PDMA},\chi_{D2}^{PDMA}\right\}<\varepsilon_{1}\right)}}\right.\\ \left.-\underset{D_{22}}{\underset{︸}{\mathrm{Pr}\left(\gamma_{SD2,k*}^{PDMA}<\varepsilon_{2}\cup \max\left\{\gamma_{SD1\leftarrow 2,k*}^{PDMA},\chi_{D2}^{PDMA}\right\}<\varepsilon_{1}\right)}}\right]\\ \times \mathrm{Pr}\left(\gamma_{SD1,k*}^{PDMA}>\varepsilon_{1}\right). \end{array}$$

Interestingly, we have the following result:

(20) $$\begin{array}{c} \mathrm{Pr}\left(\gamma_{SD2,k*}^{PDMA}<\varepsilon_{2}\right)=1-\mathrm{Pr}\left(\gamma_{SD2,k*}^{PDMA}\geq \varepsilon_{2}\right)\\ =1-\mathrm{Pr}\left({\left|g_{2,k}\right|}^{2}\geq \frac{\varepsilon_{2}\varpi \rho {\left|f_{2}\right|}^{2}+\varepsilon_{2}}{a_{2}\rho }\right)\\ =1-\sum_{k=1}^{K}\left(\begin{array}{c} K\\ k \end{array}\right){\left(-1\right)}^{k-1}\frac{a_{2}\rho \lambda_{2}}{a_{2}\rho \lambda_{2}+\varepsilon_{2}\varpi \rho k\lambda_{f_{2}}}\exp\left(-\frac{\varepsilon_{2}k}{a_{2}\rho \lambda_{2}}\right), \end{array}$$

and

(21) $$\begin{array}{c} \mathrm{Pr}\left(\gamma_{SD1,k*}^{PDMA}>\varepsilon_{1}\right)=\mathrm{Pr}\left({\left|g_{1,k}\right|}^{2}>\frac{\varepsilon_{1}\varpi \rho {\left|f_{1}\right|}^{2}+\varepsilon_{1}}{a_{1}\rho -\varepsilon_{1}a_{2}\rho }\right)\\ =\frac{1}{\lambda_{f_{1}}}\int_{0}^{\infty }\sum_{k=1}^{K}\left(\begin{array}{c} K\\ k \end{array}\right){\left(-1\right)}^{k-1}\exp\left(-\frac{\left(\varepsilon_{1}\varpi \rho x+\varepsilon_{1}\right)k}{\left(a_{1}\rho -\varepsilon_{1}a_{2}\rho \right)\lambda_{1}}-\frac{x}{\lambda_{f_{1}}}\right)dx\\ =\sum_{k=1}^{K}\left(\begin{array}{c} K\\ k \end{array}\right){\left(-1\right)}^{k-1}\vartheta_{1}. \end{array}$$

After this step, two lemmas as shown below need to be considered.

**Lemma** **1.**  *The closed-form expression of* $D_{21}$ *is calculated as*

(22) $$D_{21}=\left(1-\frac{\rho \lambda_{h_{2}}}{\rho \lambda_{h_{2}}+\varepsilon_{1}\varpi \rho \lambda_{f_{2}}}\exp\left(-\frac{\varepsilon_{1}}{\rho \lambda_{h_{2}}}\right)\right)\left(1-\sum_{k=1}^{K}\left(\begin{array}{c} K\\ k \end{array}\right){\left(-1\right)}^{k-1}\vartheta_{2}\right).$$

**Proof.**  See in Appendix B. □

**Lemma** **2.** $D_{22}$ *is computed in closed-form by*

(23) $$\begin{array}{c} D_{22}=\left(1-\sum_{k=1}^{K}\left(\begin{array}{c} K\\ k \end{array}\right){\left(-1\right)}^{k-1}\frac{a_{2}\rho \lambda_{2}}{a_{2}\rho \lambda_{2}+\varepsilon_{2}\varpi \rho k\lambda_{f_{2}}}\exp\left(-\frac{\varepsilon_{2}k}{a_{2}\rho \lambda_{2}}\right)\right)\\ \times \left(1-\sum_{k=1}^{K}\left(\begin{array}{c} K\\ k \end{array}\right){\left(-1\right)}^{k-1}\vartheta_{2}\right)\left(1-\frac{\rho \lambda_{h_{2}}}{\rho \lambda_{h_{2}}+\varepsilon_{1}\varpi \rho \lambda_{f_{2}}}\exp\left(-\frac{\varepsilon_{1}}{\rho \lambda_{h_{2}}}\right)\right). \end{array}$$

**Proof.**  See in Appendix C. □

From (20)–(23) we find the expression of $\Psi_{2}$ as below:

(24) $$\begin{array}{c} \Psi_{2}=\left[\left(1-\sum_{k=1}^{K}\left(\begin{array}{c} K\\ k \end{array}\right){\left(-1\right)}^{k-1}\frac{a_{2}\rho \lambda_{2}}{a_{2}\rho \lambda_{2}+\varepsilon_{2}\varpi \rho k\lambda_{f_{2}}}\exp\left(-\frac{\varepsilon_{2}k}{a_{2}\rho \lambda_{2}}\right)\right)\right.\\ +\left(1-\frac{\rho \lambda_{h_{2}}}{\rho \lambda_{h_{2}}+\varepsilon_{1}\varpi \rho \lambda_{f_{2}}}\exp\left(-\frac{\varepsilon_{1}}{\rho \lambda_{h_{2}}}\right)\right)\left(1-\sum_{k=1}^{K}\left(\begin{array}{c} K\\ k \end{array}\right){\left(-1\right)}^{k-1}\vartheta_{2}\right)\\ -\left(1-\sum_{k=1}^{K}\left(\begin{array}{c} K\\ k \end{array}\right){\left(-1\right)}^{k-1}\frac{a_{2}\rho \lambda_{2}}{a_{2}\rho \lambda_{2}+\varepsilon_{2}\varpi \rho k\lambda_{f_{2}}}\exp\left(-\frac{\varepsilon_{2}k}{a_{2}\rho \lambda_{2}}\right)\right)\\ \left.\times \left(1-\sum_{k=1}^{K}\left(\begin{array}{c} K\\ k \end{array}\right){\left(-1\right)}^{k-1}\vartheta_{2}\right)\left(1-\frac{\rho \lambda_{h_{2}}}{\rho \lambda_{h_{2}}+\varepsilon_{1}\varpi \rho \lambda_{f_{2}}}\exp\left(-\frac{\varepsilon_{1}}{\rho \lambda_{h_{2}}}\right)\right)\right]\\ \times \left(\sum_{k=1}^{K}\left(\begin{array}{c} K\\ k \end{array}\right){\left(-1\right)}^{k-1}\vartheta_{1}\right). \end{array}$$

From (18) and (24), the outage probability of D2D user $D_{2}$ can be examined through the formulation

(25) $$\begin{array}{c} OP_{D2-bi}=\left(1-\sum_{k=1}^{K}\left(\begin{array}{c} K\\ k \end{array}\right){\left(-1\right)}^{k-1}\frac{\lambda_{2}}{\theta k\varpi \rho \lambda_{f_{2}}+\lambda_{2}}\exp\left(-\frac{\theta k}{\lambda_{2}}\right)\right)\\ \times \left(1-\sum_{k=1}^{K}\left(\begin{array}{c} K\\ k \end{array}\right){\left(-1\right)}^{k-1}\upsilon_{1}\right)\\ +\left[\left(1-\sum_{k=1}^{K}\left(\begin{array}{c} K\\ k \end{array}\right){\left(-1\right)}^{k-1}\frac{a_{2}\rho \lambda_{2}}{a_{2}\rho \lambda_{2}+\varepsilon_{2}\varpi \rho k\lambda_{f_{2}}}\exp\left(-\frac{\varepsilon_{2}k}{a_{2}\rho \lambda_{2}}\right)\right)\right.\\ +\left(1-\frac{\rho \lambda_{h_{2}}}{\rho \lambda_{h_{2}}+\varepsilon_{1}\varpi \rho \lambda_{f_{2}}}\exp\left(-\frac{\varepsilon_{1}}{\rho \lambda_{h_{2}}}\right)\right)\left(1-\sum_{k=1}^{K}\left(\begin{array}{c} K\\ k \end{array}\right){\left(-1\right)}^{k-1}\vartheta_{2}\right)\\ -\left(1-\sum_{k=1}^{K}\left(\begin{array}{c} K\\ k \end{array}\right){\left(-1\right)}^{k-1}\frac{a_{2}\rho \lambda_{2}}{a_{2}\rho \lambda_{2}+\varepsilon_{2}\varpi \rho k\lambda_{f_{2}}}\exp\left(-\frac{\varepsilon_{2}k}{a_{2}\rho \lambda_{2}}\right)\right)\\ \left.\times \left(1-\sum_{k=1}^{K}\left(\begin{array}{c} K\\ k \end{array}\right){\left(-1\right)}^{k-1}\vartheta_{2}\right)\left(1-\frac{\rho \lambda_{h_{2}}}{\rho \lambda_{h_{2}}+\varepsilon_{1}\varpi \rho \lambda_{f_{2}}}\exp\left(-\frac{\varepsilon_{1}}{\rho \lambda_{h_{2}}}\right)\right)\right]\\ \times \left(\sum_{k=1}^{K}\left(\begin{array}{c} K\\ k \end{array}\right){\left(-1\right)}^{k-1}\vartheta_{1}\right). \end{array}$$

## 4. Analysis On Asymptotic Outage Probability

Based on the previous results, an asymptotic analysis for both $D_{1}$ and $D_{2}$ will be carried out to evaluate the outage behavior, i.e., $OP_{D1-bi}$ and $OP_{D2-bi}$, respectively. Particularly, the following expressions are provide insight observation for the proposed system in the high SNR regime.

### 4.1. Asymptotic Outage Probability at D2D User 1

Based on the above analytical results in (12), by using $e^{-x}\approx 1-x$ the asymptotic outage probability of D2D User 1 with is given by

(26) $$\begin{array}{c} OP_{D1-asym}=\left(1-\sum_{k=1}^{K}\left(\begin{array}{c} K\\ k \end{array}\right){\left(-1\right)}^{k-1}\frac{\left(a_{1}-\varepsilon_{1}a_{2}\right)\lambda_{1}}{k\varepsilon_{1}\varpi \lambda_{f_{1}}+\left(a_{1}-\varepsilon_{1}a_{2}\right)\lambda_{1}}\left(1-\frac{\varepsilon_{1}k}{\left(a_{1}\rho -\varepsilon_{1}a_{2}\rho \right)\lambda_{1}}\right)\right)\\ \times \left(1-\sum_{k=1}^{K}\left(\begin{array}{c} K\\ k \end{array}\right){\left(-1\right)}^{k-1}\frac{\left(a_{1}-\varepsilon_{1}a_{2}\right)\lambda_{2}}{\left(a_{1}-\varepsilon_{1}a_{2}\right)\lambda_{2}+\varepsilon_{1}\varpi k\lambda_{f_{2}}}\left(1-\frac{\varepsilon_{1}k}{\left(a_{1}\rho -\varepsilon_{1}a_{2}\rho \right)\lambda_{2}}\right)\right)\\ +\left(1-\sum_{k=1}^{K}\left(\begin{array}{c} K\\ k \end{array}\right){\left(-1\right)}^{k-1}\frac{\left(a_{1}-\varepsilon_{1}a_{2}\right)\lambda_{1}}{\left(a_{1}-\varepsilon_{1}a_{2}\right)\lambda_{1}+\varepsilon_{1}\varpi k\lambda_{f_{1}}}\left(1-\frac{\varepsilon_{1}k}{\left(a_{1}\rho -\varepsilon_{1}a_{2}\rho \right)\lambda_{1}}\right)\right)\\ \times \left(1-\frac{\lambda_{h_{1}}}{\lambda_{h_{1}}+\varepsilon_{1}\varpi \lambda_{f_{1}}}\left(1-\frac{\varepsilon_{1}}{\rho \lambda_{h_{1}}}\right)\right)\sum_{k=1}^{K}\left(\begin{array}{c} K\\ k \end{array}\right){\left(-1\right)}^{k-1}\frac{\left(a_{1}-\varepsilon_{1}a_{2}\right)\lambda_{2}}{\left(a_{1}-\varepsilon_{1}a_{2}\right)\lambda_{2}+\varepsilon_{1}\varpi k\lambda_{f_{2}}}\left(1-\frac{\varepsilon_{1}k}{\left(a_{1}\rho -\varepsilon_{1}a_{2}\rho \right)\lambda_{2}}\right). \end{array}$$

To look lower bound, when ρ→∞, the asymptotic outage probability of D2D User 1 with is determined by

(27) $$\begin{array}{c} OP_{D1-floor}=\left(1-\sum_{k=1}^{K}\left(\begin{array}{c} K\\ k \end{array}\right){\left(-1\right)}^{k-1}\frac{\left(a_{1}-\varepsilon_{1}a_{2}\right)\lambda_{1}}{k\varepsilon_{1}\varpi \lambda_{f_{1}}+\left(a_{1}-\varepsilon_{1}a_{2}\right)\lambda_{1}}\right)\\ \times \left(1-\sum_{k=1}^{K}\left(\begin{array}{c} K\\ k \end{array}\right){\left(-1\right)}^{k-1}\frac{\left(a_{1}-\varepsilon_{1}a_{2}\right)\lambda_{2}}{\left(a_{1}-\varepsilon_{1}a_{2}\right)\lambda_{2}+\varepsilon_{1}\varpi k\lambda_{f_{2}}}\right)\\ +\left(1-\sum_{k=1}^{K}\left(\begin{array}{c} K\\ k \end{array}\right){\left(-1\right)}^{k-1}\frac{\left(a_{1}-\varepsilon_{1}a_{2}\right)\lambda_{1}}{\left(a_{1}-\varepsilon_{1}a_{2}\right)\lambda_{1}+\varepsilon_{1}\varpi k\lambda_{f_{1}}}\right)\\ \times \left(1-\frac{\lambda_{h_{1}}}{\lambda_{h_{1}}+\varepsilon_{1}\varpi \lambda_{f_{1}}}\right)\sum_{k=1}^{K}\left(\begin{array}{c} K\\ k \end{array}\right){\left(-1\right)}^{k-1}\frac{\left(a_{1}-\varepsilon_{1}a_{2}\right)\lambda_{2}}{\left(a_{1}-\varepsilon_{1}a_{2}\right)\lambda_{2}+\varepsilon_{1}\varpi k\lambda_{f_{2}}}. \end{array}$$

### 4.2. Asymptotic Outage Probability at D2D User 2

Similar to the derivation of $OP_{D1-asym}$, an asymptotic outage expression for $OP_{D2-bi}$. It is noted that the related exact expression is presented in (25), now it can be derived as

(28) $$\begin{array}{c} OP_{D2-asym}=\left(1-\sum_{k=1}^{K}\left(\begin{array}{c} K\\ k \end{array}\right){\left(-1\right)}^{k-1}\frac{\lambda_{2}}{\theta k\varpi \rho \lambda_{f_{2}}+\lambda_{2}}\left(1-\frac{\theta k}{\lambda_{2}}\right)\right)\\ \times \left(1-\sum_{k=1}^{K}\left(\begin{array}{c} K\\ k \end{array}\right){\left(-1\right)}^{k-1}\frac{\left(a_{1}-\varepsilon_{2}a_{2}\right)\lambda_{1}}{\left(a_{1}-\varepsilon_{2}a_{2}\right)\lambda_{1}+\varepsilon_{2}\varpi k\lambda_{f_{1}}}\left(1-\frac{\varepsilon_{2}k}{\left(a_{1}\rho -\varepsilon_{2}a_{2}\rho \right)\lambda_{1}}\right)\right)\\ +\left[\left(1-\sum_{k=1}^{K}\left(\begin{array}{c} K\\ k \end{array}\right){\left(-1\right)}^{k-1}\frac{a_{2}\lambda_{2}}{a_{2}\lambda_{2}+\varepsilon_{2}\varpi k\lambda_{f_{2}}}\left(1-\frac{\varepsilon_{2}k}{a_{2}\rho \lambda_{2}}\right)\right)\right.\\ +\left(1-\frac{\lambda_{h_{2}}}{\lambda_{h_{2}}+\varepsilon_{1}\varpi \lambda_{f_{2}}}\left(1-\frac{\varepsilon_{1}}{\rho \lambda_{h_{2}}}\right)\right)\left(1-\sum_{k=1}^{K}\left(\begin{array}{c} K\\ k \end{array}\right){\left(-1\right)}^{k-1}\frac{\left(a_{1}-\varepsilon_{1}a_{2}\right)\lambda_{2}}{\left(a_{1}-\varepsilon_{1}a_{2}\right)\lambda_{2}+\varepsilon_{1}\varpi k\lambda_{f_{2}}}\left(1-\frac{\varepsilon_{1}k}{\left(a_{1}\rho -\varepsilon_{1}a_{2}\rho \right)\lambda_{2}}\right)\right)\\ -\left(1-\sum_{k=1}^{K}\left(\begin{array}{c} K\\ k \end{array}\right){\left(-1\right)}^{k-1}\frac{a_{2}\lambda_{2}}{a_{2}\lambda_{2}+\varepsilon_{2}\varpi k\lambda_{f_{2}}}\left(1-\frac{\varepsilon_{2}k}{a_{2}\rho \lambda_{2}}\right)\right)\\ \times \left.\left(1-\sum_{k=1}^{K}\left(\begin{array}{c} K\\ k \end{array}\right){\left(-1\right)}^{k-1}\frac{\left(a_{1}-\varepsilon_{1}a_{2}\right)\lambda_{2}}{\left(a_{1}-\varepsilon_{1}a_{2}\right)\lambda_{2}+\varepsilon_{1}\varpi k\lambda_{f_{2}}}\left(1-\frac{\varepsilon_{1}k}{\left(a_{1}\rho -\varepsilon_{1}a_{2}\rho \right)\lambda_{2}}\right)\right)\left(1-\frac{\lambda_{h_{2}}}{\lambda_{h_{2}}+\varepsilon_{1}\varpi \lambda_{f_{2}}}\left(1-\frac{\varepsilon_{1}}{\rho \lambda_{h_{2}}}\right)\right)\right]\\ \times \left(\sum_{k=1}^{K}\left(\begin{array}{c} K\\ k \end{array}\right){\left(-1\right)}^{k-1}\frac{\left(a_{1}-\varepsilon_{1}a_{2}\right)\lambda_{1}}{\left(a_{1}-\varepsilon_{1}a_{2}\right)\lambda_{1}+\varepsilon_{1}\varpi k\lambda_{f_{1}}}\left(1-\frac{\varepsilon_{1}k}{\left(a_{1}\rho -\varepsilon_{1}a_{2}\rho \right)\lambda_{1}}\right)\right), \end{array}$$

and with regard to lower bound, it can be obtained lower bound of user $D_{2}$ as

(29) $$\begin{array}{c} OP_{D2-floor}=\left(1-\sum_{k=1}^{K}\left(\begin{array}{c} K\\ k \end{array}\right){\left(-1\right)}^{k-1}\frac{\lambda_{2}}{\theta k\varpi \rho \lambda_{f_{2}}+\lambda_{2}}\right)\left(1-\sum_{k=1}^{K}\left(\begin{array}{c} K\\ k \end{array}\right){\left(-1\right)}^{k-1}\frac{\left(a_{1}-\varepsilon_{2}a_{2}\right)\lambda_{1}}{\left(a_{1}-\varepsilon_{2}a_{2}\right)\lambda_{1}+\varepsilon_{2}\varpi k\lambda_{f_{1}}}\right)\\ +\left[\left(1-\sum_{k=1}^{K}\left(\begin{array}{c} K\\ k \end{array}\right){\left(-1\right)}^{k-1}\frac{a_{2}\lambda_{2}}{a_{2}\lambda_{2}+\varepsilon_{2}\varpi k\lambda_{f_{2}}}\right)\right.\\ +\left(1-\frac{\lambda_{h_{2}}}{\lambda_{h_{2}}+\varepsilon_{1}\varpi \lambda_{f_{2}}}\right)\left(1-\sum_{k=1}^{K}\left(\begin{array}{c} K\\ k \end{array}\right){\left(-1\right)}^{k-1}\frac{\left(a_{1}-\varepsilon_{1}a_{2}\right)\lambda_{2}}{\left(a_{1}-\varepsilon_{1}a_{2}\right)\lambda_{2}+\varepsilon_{1}\varpi k\lambda_{f_{2}}}\right)\\ -\left(1-\sum_{k=1}^{K}\left(\begin{array}{c} K\\ k \end{array}\right){\left(-1\right)}^{k-1}\frac{a_{2}\lambda_{2}}{a_{2}\lambda_{2}+\varepsilon_{2}\varpi k\lambda_{f_{2}}}\right)\\ \times \left.\left(1-\sum_{k=1}^{K}\left(\begin{array}{c} K\\ k \end{array}\right){\left(-1\right)}^{k-1}\frac{\left(a_{1}-\varepsilon_{1}a_{2}\right)\lambda_{2}}{\left(a_{1}-\varepsilon_{1}a_{2}\right)\lambda_{2}+\varepsilon_{1}\varpi k\lambda_{f_{2}}}\right)\left(1-\frac{\lambda_{h_{2}}}{\lambda_{h_{2}}+\varepsilon_{1}\varpi \lambda_{f_{2}}}\right)\right]\\ \times \left(\sum_{k=1}^{K}\left(\begin{array}{c} K\\ k \end{array}\right){\left(-1\right)}^{k-1}\frac{\left(a_{1}-\varepsilon_{1}a_{2}\right)\lambda_{1}}{\left(a_{1}-\varepsilon_{1}a_{2}\right)\lambda_{1}+\varepsilon_{1}\varpi k\lambda_{f_{1}}}\right). \end{array}$$

**Remark** **1.**  *These approximate performances provide easy way to evaluate system performance rather than complex manner of derived expressions in term of outage probability. It is expected that these approximate expressions exhibits corresponding curves in simulation and they will match with exact curves achieved by analytical expressions presented in Section 3.*

## 5. Numerical Results

In this section, numerical examples are performed to verify the outage performance of the downlink MISO PDMA network under Rayleigh fading channels with FD scheme. We denote $d_{1},d_{2}$ as distances between the BS and the first D2D user and the second one, repetitively. Such distance is normalized as unit. Moreover, Monte Carlo simulation is run in $10^{6}$ times to compare with analytical results as proved in previous section.

**Figure 2:**
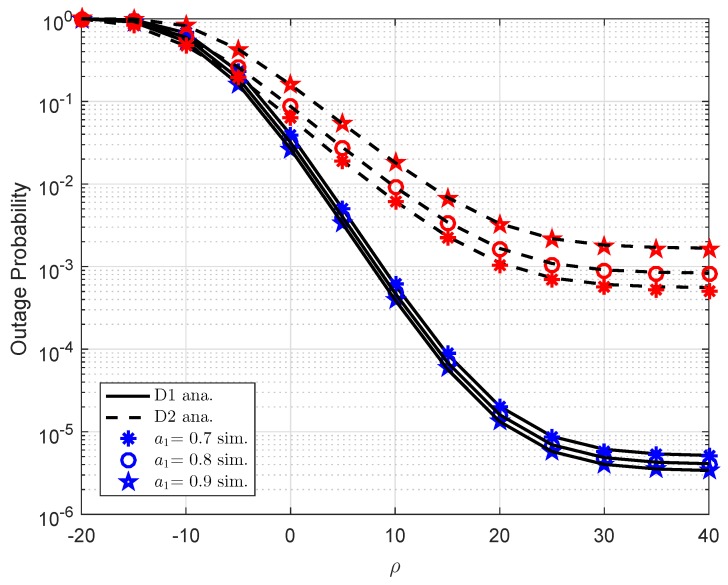
Outage performance of $D_{1}$ and $D_{2}$ versus ρ as varying $a_{1}$.

**Figure 3:**
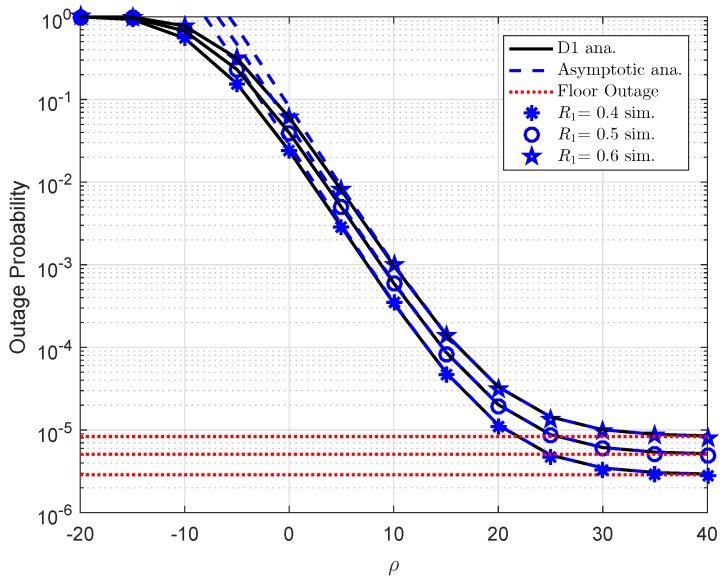
Outage performance of $D_{1}$ as varying $R_{1}$.

In Figure 2, the outage probability versus transmit SNR at the BS ρ is presented in different power allocation parameters. We assume the distance between BS and $D_{1}$ is $d_{1}=0.4$, path loss exponent is α=2, channel gain $\lambda_{2}=d_{2}^{\left(-\alpha \right)}$, while $d_{2}=0.2$, $\lambda_{h_{1}}=\lambda_{h_{2}}=1,\lambda_{f_{1}}=\lambda_{f_{2}}=0.01$, the number of antenna at BS is K=1. As a clear observation, the exact analytical results and simulation results are in excellent agreement, and the outage probability will be constant at high-SNR regimes. Moreover, as the transmit SNR increases, the outage probability decreases. Another important observation is that the outage probability for User 2 $D_{2}$ outperforms User 1 $D_{1}$. Figure 3 shows outage performance for user $D_{1}$. The parameters for this case are $a_{1}=0.7,\lambda_{1}=d_{1}^{\left(-\alpha \right)},d_{1}=0.4,\alpha =2,\lambda_{2}=d_{2}^{\left(-\alpha \right)},d_{2}=0.2,\lambda_{h_{1}}=\lambda_{h_{2}}=1,\lambda_{f_{1}}=\lambda_{f_{2}}=0.01,K=1$. It can be seen that lower target rate $R_{1}$ results in better outage performance. It is intuitively that floor outage values match with analytical curves at high ρ. Such observation confirms our analysis on finding lower bound of outage probability. While asymptotic lines also match with exact lines at several points within the range of considered transmit SNR at the BS.

**Figure 4:**
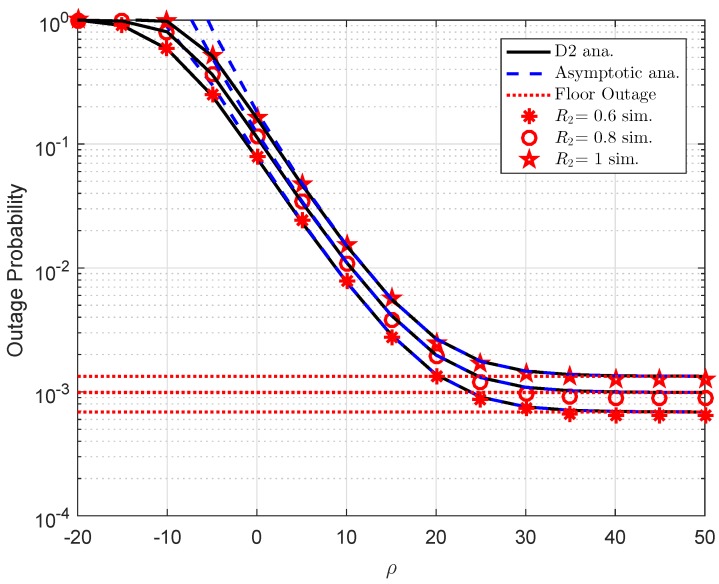
Outage performance of $D_{2}$ versus ρ as varying $R_{2}$.

In Figure 4, the outage probabilities are shown as a function of the transmit SNR. Reported from the impact of target rate $R_{2}$, there is a decrease in outage probability for such user as change to lower level of $R_{2}$. This figure requires several parameters as $a_{1}=0.7,R_{1}=0.5,\lambda_{1}=d_{1}^{\left(-\alpha \right)},d_{1}=0.4,\alpha =2,\lambda_{2}=d_{2}^{\left(-\alpha \right)},d_{2}=0.2,\lambda_{h_{1}}=\lambda_{h_{2}}=1,\lambda_{f_{1}}=\lambda_{f_{2}}=0.01,K=1$. Similar trends with Figure 3 in terms of approximate and floor value of outage for $D_{2}$ can be observed in this figure.

**Figure 5:**
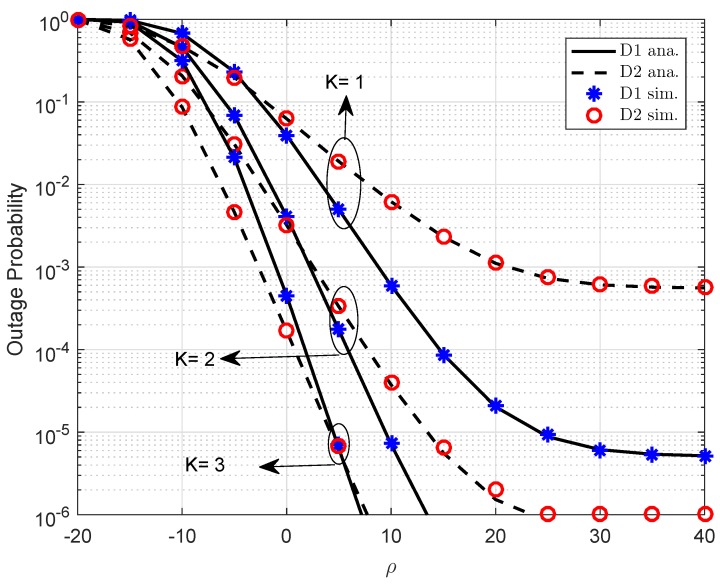
Outage performance of $D_{1}$ and $D_{2}$ as varying *K*.

Figure 5 plots the outage probability versus SNR with the different numbers of transmit antennas at the BS (other parameters as declarations in Figure 5 as $a_{1}=0.7,R_{1}=0.5,\lambda_{1}=d_{1}^{\left(-\alpha \right)},d_{1}=0.4.\alpha =2,\lambda_{2}=d_{2}^{\left(-\alpha \right)},d_{2}=0.2,\lambda_{h_{1}}=\lambda_{h_{2}}=1,\lambda_{f_{1}}=\lambda_{f_{2}}=0.01$). More antennas at the BS indicates better outage probability in such PDMA. K=3 case provides the best performance and an important observation in this study.

**Figure 6:**
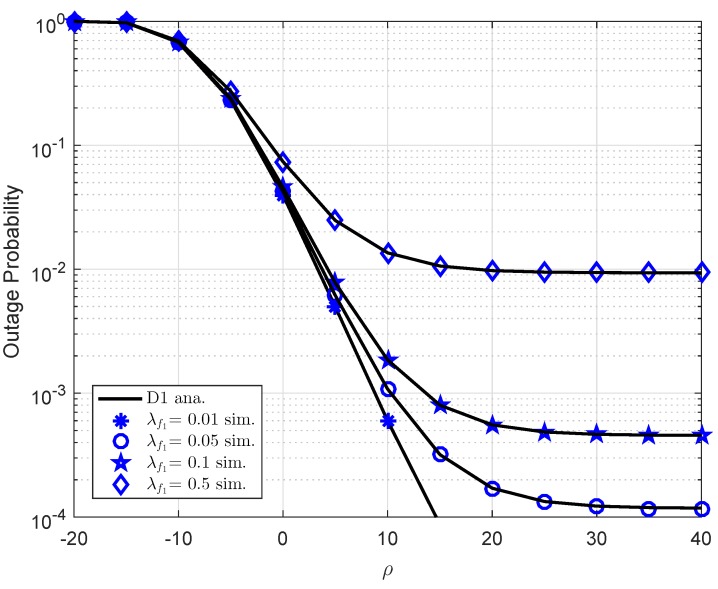
Outage behavior of user $D_{1}$ versus ρ as varying $\lambda_{f_{1}}$.

**Figure 7:**
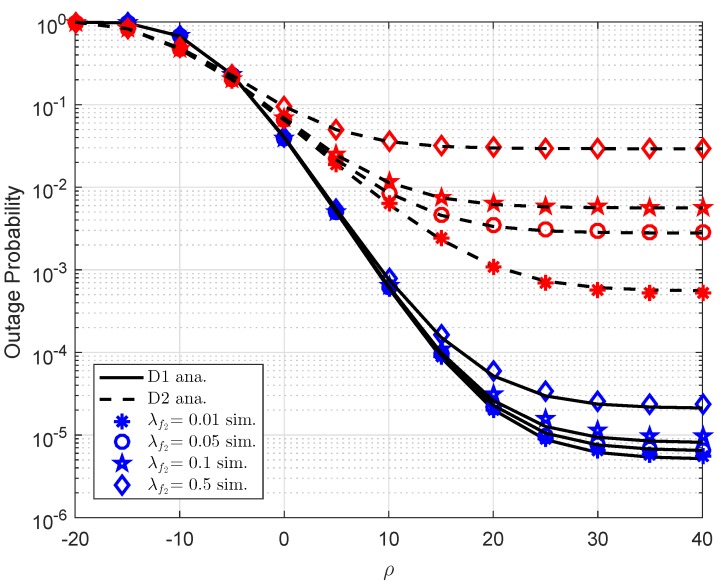
Consideration on outage of $D_{1}$ and $D_{2}$ versus ρ as varying $\lambda_{f_{2}}$.

Due to self-interference related to FD scheme at users, it need be considered performance of user $D_{1}$ in four cases of $\lambda_{f1}$ as observation in Figure 6. In this case, we set $a_{1}=0.7,R_{1}=0.5,\lambda_{1}=d_{1}^{\left(-\alpha \right)},d_{1}=0.4,\alpha =2,\lambda_{2}=d_{2}^{\left(-\alpha \right)},d_{2}=0.2,\lambda_{h_{1}}=\lambda_{h_{2}}=1,K=1$ for both Figure 6 and Figure 7. It is noted that $\lambda_{f2}=0.01,\lambda_{f_{1}}=0.01$ for Figure 6, Figure 7, respectively. Obviously, strong self-interference makes outage performance worse. The main reason is that achievable SNR will be smaller as existence of self-interference and hence outage event easily happens. Similarly, performance of $D_{2}$ in Figure 7 is changed as varying $\lambda_{f_{2}}$.

## 6. Conclusions

This paper analytically investigated the impact of number of transmit antennas at the BS on outage performance of each D2D user in the MISO PDMA. Closed-form analytical expressions for the outage probability were obtained. Our theoretical analysis indicated that the outage performance gap between two D2D users exists due to different power allocation factors given. The best performance can be raised at a higher number of transmit antennas at the BS. Furthermore, we observed that target rates have only a small impact on outage performance.

## Appendix A.

**Proof** **of** **Proposition** **1.** The terms $B_{1}$ and $B_{2}$ can be calculated with the help of (2) and (3). The former becomes

(A1) $$\begin{array}{c} B_{1}=\mathrm{Pr}\left(\gamma_{SD1,k*}^{PDMA}<\varepsilon_{1},\gamma_{SD1\leftarrow 2,k*}^{PDMA}<\varepsilon_{1}\right)\\ =\underset{B_{11}}{\underset{︸}{\left(1-\mathrm{Pr}\left(\gamma_{SD1,k*}^{PDMA}\geq \varepsilon_{1}\right)\right)}}\underset{B_{12}}{\underset{︸}{\left(1-\mathrm{Pr}\left(\gamma_{SD1\leftarrow 2,k*}^{PDMA}\geq \varepsilon_{1}\right)\right)}} \end{array}.$$

Therefore, $B_{11}$ can be expressed as

(A2) $$\begin{array}{c} B_{11}=1-\mathrm{Pr}\left(\gamma_{SD1,k}^{PDMA}\geq \varepsilon_{1}\right)\\ =1-\mathrm{Pr}\left({\left|g_{1,k}\right|}^{2}\geq \frac{\varepsilon_{1}\varpi \rho {\left|f_{1}\right|}^{2}+\varepsilon_{1}}{a_{1}\rho -\varepsilon_{1}a_{2}\rho }\right)\\ =1-\frac{1}{\lambda_{f_{1}}}\int_{0}^{\infty }\sum_{k=1}^{K}\left(\begin{array}{c} K\\ k \end{array}\right){\left(-1\right)}^{k-1}\exp\left(-\frac{\left(\varepsilon_{1}\varpi \rho x+\varepsilon_{1}\right)k}{\left(a_{1}\rho -\varepsilon_{1}a_{2}\rho \right)\lambda_{1}}-\frac{x}{\lambda_{f_{1}}}\right)dx\\ =1-\sum_{k=1}^{K}\left(\begin{array}{c} K\\ k \end{array}\right){\left(-1\right)}^{k-1}\vartheta_{1}, \end{array}$$

where $\vartheta_{1}=\frac{\left(a_{1}\rho -\varepsilon_{1}a_{2}\rho \right)\lambda_{1}}{k\varepsilon_{1}\varpi \rho \lambda_{f_{1}}+\left(a_{1}\rho -\varepsilon_{1}a_{2}\rho \right)\lambda_{1}}\exp\left(-\frac{\varepsilon_{1}k}{\left(a_{1}\rho -\varepsilon_{1}a_{2}\rho \right)\lambda_{1}}\right)$. It is noted that $\lambda_{1}$ is average channel gain of $g_{1,k}$, then $\lambda_{2}$ is average channel gain of $g_{2,k}$, $\lambda_{f1},\lambda_{f2}$ are average channel gain of $f_{1},f_{2}$, respectively; $\lambda_{h1},\lambda_{h2}$ are average channel gains of $h_{1},h_{2}$, respectively. Similarly, $B_{12}$ can be expressed by

(A3) $$\begin{array}{c} B_{12}=1-\mathrm{Pr}\left(\gamma_{SD1\leftarrow 2,k}^{PDMA}\geq \varepsilon_{1}\right)\\ =1-\mathrm{Pr}\left({\left|g_{2,k}\right|}^{2}\geq \frac{\varepsilon_{1}\varpi \rho {\left|f_{2}\right|}^{2}+\varepsilon_{1}}{a_{1}\rho -\varepsilon_{1}a_{2}\rho }\right)\\ =1-\frac{1}{\lambda_{f_{2}}}\int_{0}^{\infty }\sum_{k=1}^{K}\left(\begin{array}{c} K\\ k \end{array}\right){\left(-1\right)}^{k-1}\exp\left(-\frac{\left(\varepsilon_{1}\varpi \rho y+\varepsilon_{1}\right)k}{\left(a_{1}\rho -\varepsilon_{1}a_{2}\rho \right)\lambda_{2}}-\frac{y}{\lambda_{f_{2}}}\right)dy\\ =1-\sum_{k=1}^{K}\left(\begin{array}{c} K\\ k \end{array}\right){\left(-1\right)}^{k-1}\vartheta_{2}, \end{array}$$

where $\vartheta_{2}=\frac{\left(a_{1}\rho -\varepsilon_{1}a_{2}\rho \right)\lambda_{2}}{\left(a_{1}\rho -\varepsilon_{1}a_{2}\rho \right)\lambda_{2}+\varepsilon_{1}\varpi \rho k\lambda_{f_{2}}}\exp\left(-\frac{\varepsilon_{1}k}{\left(a_{1}\rho -\varepsilon_{1}a_{2}\rho \right)\lambda_{2}}\right)$. From (A2) and (A3) we find the expression $B_{1}$ as

(A4) $$B_{1}=\left(1-\sum_{k=1}^{K}\left(\begin{array}{c} K\\ k \end{array}\right){\left(-1\right)}^{k-1}\vartheta_{1}\right)\left(1-\sum_{k=1}^{K}\left(\begin{array}{c} K\\ k \end{array}\right){\left(-1\right)}^{k-1}\vartheta_{2}\right).$$

In similar way, $B_{2}$ can be computed as

(A5) $$\begin{array}{c} B_{2}=\mathrm{Pr}\left(\max\left\{\gamma_{SD1,k*}^{PDMA},\chi_{D1}^{PDMA}\right\}<\varepsilon_{1},\gamma_{SD1\leftarrow 2,k*}^{PDMA}>\varepsilon_{1}\right)\\ =\mathrm{Pr}\left(\gamma_{SD1,k*}^{PDMA}<\varepsilon_{1}\right)\mathrm{Pr}\left(\chi_{D1}^{PDMA}<\varepsilon_{1}\right)\mathrm{Pr}\left(\gamma_{SD1\leftarrow 2,k*}^{PDMA}>\varepsilon_{1}\right)\\ =\underset{B_{21}}{\underset{︸}{\left(1-\mathrm{Pr}\left(\gamma_{SD1,k*}^{PDMA}\geq \varepsilon_{1}\right)\right)}}\underset{B_{22}}{\underset{︸}{\left(1-\mathrm{Pr}\left(\chi_{D1}^{PDMA}\geq \varepsilon_{1}\right)\right)}}\\ \times \underset{B_{23}}{\underset{︸}{\mathrm{Pr}\left(\gamma_{SD1\leftarrow 2,k*}^{PDMA}>\varepsilon_{1}\right)}}. \end{array}$$

In this step, $B_{21}$ is formulated as

(A6) $$\begin{array}{c} B_{21}=\mathrm{Pr}\left(\gamma_{SD1,k*}^{PDMA}\geq \varepsilon_{1}\right)=\mathrm{Pr}\left({\left|g_{1,k}\right|}^{2}\geq \frac{\varepsilon_{1}\varpi \rho {\left|f_{1}\right|}^{2}+\varepsilon_{1}}{a_{1}\rho -\varepsilon_{1}a_{2}\rho }\right)\\ =\frac{1}{\lambda_{f_{1}}}\int_{0}^{\infty }\sum_{k=1}^{K}\left(\begin{array}{c} K\\ k \end{array}\right){\left(-1\right)}^{k-1}\exp\left(-\frac{\left(\varepsilon_{1}\varpi \rho x+\varepsilon_{1}\right)k}{\left(a_{1}\rho -\varepsilon_{1}a_{2}\rho \right)\lambda_{1}}-\frac{x}{\lambda_{f_{1}}}\right)dx\\ =\sum_{k=1}^{K}\left(\begin{array}{c} K\\ k \end{array}\right){\left(-1\right)}^{k-1}\vartheta_{1}. \end{array}$$

Then, $B_{22}$ is calculated as

(A7) $$\begin{array}{c} B_{22}=\mathrm{Pr}\left(\chi_{D1}^{PDMA}\geq \varepsilon_{1}\right)=\mathrm{Pr}\left({\left|h_{1}\right|}^{2}\geq \frac{\varepsilon_{1}\varpi \rho {\left|f_{1}\right|}^{2}+\varepsilon_{1}}{\rho }\right)\\ =\int_{0}^{\infty }\exp\left(-\frac{\varepsilon_{1}\varpi \rho x+\varepsilon_{1}}{\rho \lambda_{h_{1}}}\right)\frac{1}{\lambda_{f_{1}}}\exp\left(-\frac{x}{\lambda_{f_{1}}}\right)dx\\ =\frac{1}{\lambda_{f_{1}}}\exp\left(-\frac{\varepsilon_{1}}{\rho \lambda_{h_{1}}}\right)\int_{0}^{\infty }\exp\left(-\left(\frac{\varepsilon_{1}\varpi \rho }{\rho \lambda_{h_{1}}}+\frac{1}{\lambda_{f_{1}}}\right)x\right)dx\\ =\frac{\rho \lambda_{h_{1}}}{\rho \lambda_{h_{1}}+\varepsilon_{1}\varpi \rho \lambda_{f_{1}}}\exp\left(-\frac{\varepsilon_{1}}{\rho \lambda_{h_{1}}}\right). \end{array}$$

Similarly, $B_{23}$ is calculated as

(A8) $$\begin{array}{c} B_{23}=\mathrm{Pr}\left(\gamma_{SD1\leftarrow 2,k*}^{PDMA}>\varepsilon_{1}\right)=\mathrm{Pr}\left({\left|g_{2,k}\right|}^{2}>\frac{\varepsilon_{1}\varpi \rho {\left|f_{2}\right|}^{2}+\varepsilon_{1}}{a_{1}\rho -\varepsilon_{1}a_{2}\rho }\right)\\ =\frac{1}{\lambda_{f_{2}}}\int_{0}^{\infty }\sum_{k=1}^{K}\left(\begin{array}{c} K\\ k \end{array}\right){\left(-1\right)}^{k-1}\exp\left(-\frac{\left(\varepsilon_{1}\varpi \rho y+\varepsilon_{1}\right)k}{\left(a_{1}\rho -\varepsilon_{1}a_{2}\rho \right)\lambda_{2}}-\frac{y}{\lambda_{f_{2}}}\right)dy\\ =\sum_{k=1}^{K}\left(\begin{array}{c} K\\ k \end{array}\right){\left(-1\right)}^{k-1}\vartheta_{2}. \end{array}$$

From (A6), (A7) and (A8) we find the expression $B_{2}$ to be

(A9) $$\begin{array}{c} B_{2}=\left(1-\sum_{k=1}^{K}\left(\begin{array}{c} K\\ k \end{array}\right){\left(-1\right)}^{k-1}\vartheta_{1}\right)\\ \times \left(1-\frac{\rho \lambda_{h_{1}}}{\rho \lambda_{h_{1}}+\varepsilon_{1}\varpi \rho \lambda_{f_{1}}}\exp\left(-\frac{\varepsilon_{1}}{\rho \lambda_{h_{1}}}\right)\right)\sum_{k=1}^{K}\left(\begin{array}{c} K\\ k \end{array}\right){\left(-1\right)}^{k-1}\vartheta_{2}. \end{array}$$

This completes the proof of Proposition 1. □

## Appendix B.

**Proof** **of** **Lemma** **1.**

(A10) $$D_{21}=\underset{\kappa_{1}}{\underset{︸}{\left(1-\mathrm{Pr}\left(\chi_{D2}^{PDMA}\geq \varepsilon_{1}\right)\right)}}\underset{\kappa_{2}}{\underset{︸}{\left(1-\mathrm{Pr}\left(\gamma_{SD1\leftarrow 2,k}^{PDMA}\geq \varepsilon_{1}\right)\right)}}.$$

Here, $\kappa_{1}$ can be calculated as

(A11) $$\begin{array}{c} \kappa_{1}=1-\mathrm{Pr}\left(\chi_{D2}^{PDMA}\geq \varepsilon_{1}\right)\\ =1-\mathrm{Pr}\left({\left|h_{2}\right|}^{2}\geq \frac{\varepsilon_{1}\varpi \rho {\left|f_{2}\right|}^{2}+\varepsilon_{1}}{\rho }\right)\\ =1-\frac{1}{\lambda_{f_{2}}}\int_{0}^{\infty }\exp\left(-\frac{\varepsilon_{1}\varpi \rho y+\varepsilon_{1}}{\rho \lambda_{h_{2}}}-\frac{y}{\lambda_{f_{2}}}\right)dy\\ =1-\frac{\rho \lambda_{h_{2}}}{\rho \lambda_{h_{2}}+\varepsilon_{1}\varpi \rho \lambda_{f_{2}}}\exp\left(-\frac{\varepsilon_{1}}{\rho \lambda_{h_{2}}}\right). \end{array}$$

Similarly, $\kappa_{2}$ can be calculated as

(A12) $$\begin{array}{c} \kappa_{2}=1-\mathrm{Pr}\left(\gamma_{SD1\leftarrow 2,k}^{PDMA}\geq \varepsilon_{1}\right)\\ =1-\mathrm{Pr}\left({\left|g_{2,k}\right|}^{2}\geq \frac{\varepsilon_{1}\varpi \rho {\left|f_{2}\right|}^{2}+\varepsilon_{1}}{a_{1}\rho -\varepsilon_{1}a_{2}\rho }\right)\\ =1-\frac{1}{\lambda_{f_{2}}}\int_{0}^{\infty }\sum_{k=1}^{K}\left(\begin{array}{c} K\\ k \end{array}\right){\left(-1\right)}^{k-1}\exp\left(-\frac{\left(\varepsilon_{1}\varpi \rho y+\varepsilon_{1}\right)k}{\left(a_{1}\rho -\varepsilon_{1}a_{2}\rho \right)\lambda_{2}}-\frac{y}{\lambda_{f_{2}}}\right)dy\\ =1-\sum_{k=1}^{K}\left(\begin{array}{c} K\\ k \end{array}\right){\left(-1\right)}^{k-1}\vartheta_{2}. \end{array}$$

This completes the proof of Lemma 1. □

## Appendix C.

**Proof** **of** **Lemma** **2.** By definition, we have following outage probability:

(A13) $$\begin{array}{c} D_{22}=\mathrm{Pr}\left(\gamma_{SD2,k*}^{PDMA}<\varepsilon_{2}\cup \max\left\{\gamma_{SD1\leftarrow 2,k*}^{PDMA},\chi_{D2}^{PDMA}\right\}<\varepsilon_{1}\right)\\ =\underset{\Xi_{1}}{\underset{︸}{\left(1-\mathrm{Pr}\left(\gamma_{SD2,k}^{PDMA}\geq \varepsilon_{2}\right)\right)}}\underset{\Xi_{2}}{\underset{︸}{\left(1-\mathrm{Pr}\left(\gamma_{SD1\leftarrow 2,k}^{PDMA}\geq \varepsilon_{1}\right)\right)}}\\ \times \underset{\Xi_{3}}{\underset{︸}{\left(1-\mathrm{Pr}\left(\chi_{D2}^{PDMA}\geq \varepsilon_{1}\right)\right)}}. \end{array}$$

Firstly, $\Xi_{1}$ can be calculated as

(A14) $$\begin{array}{c} \Xi_{1}=1-\mathrm{Pr}\left(\gamma_{SD2,k}^{PDMA}\geq \varepsilon_{2}\right)\\ =1-\mathrm{Pr}\left({\left|g_{2,k}\right|}^{2}\geq \frac{\varepsilon_{2}\varpi \rho {\left|f_{2}\right|}^{2}+\varepsilon_{2}}{a_{2}\rho }\right)\\ =1-\frac{1}{\lambda_{f_{2}}}\int_{0}^{\infty }\sum_{k=1}^{K}\left(\begin{array}{c} K\\ k \end{array}\right){\left(-1\right)}^{k-1}\exp\left(-\frac{\left(\varepsilon_{2}\varpi \rho y+\varepsilon_{2}\right)k}{a_{2}\rho \lambda_{2}}-\frac{y}{\lambda_{f_{2}}}\right)dy\\ =1-\sum_{k=1}^{K}\left(\begin{array}{c} K\\ k \end{array}\right){\left(-1\right)}^{k-1}\frac{a_{2}\rho \lambda_{2}}{a_{2}\rho \lambda_{2}+\varepsilon_{2}\varpi \rho k\lambda_{f_{2}}}\exp\left(-\frac{\varepsilon_{2}k}{a_{2}\rho \lambda_{2}}\right). \end{array}$$

Similarly, $\Xi_{2}$ can be calculated as

(A15) $$\begin{array}{c} \Xi_{2}=1-\mathrm{Pr}\left(\gamma_{SD1\leftarrow 2,k}^{PDMA}\geq \varepsilon_{1}\right)\\ =1-\mathrm{Pr}\left({\left|g_{2,k}\right|}^{2}\geq \frac{\varepsilon_{1}\varpi \rho {\left|f_{2}\right|}^{2}+\varepsilon_{1}}{a_{1}\rho -\varepsilon_{1}a_{2}\rho }\right)\\ =1-\frac{1}{\lambda_{f_{2}}}\int_{0}^{\infty }\sum_{k=1}^{K}\left(\begin{array}{c} K\\ k \end{array}\right){\left(-1\right)}^{k-1}\exp\left(-\frac{\left(\varepsilon_{1}\varpi \rho y+\varepsilon_{1}\right)k}{\left(a_{1}\rho -\varepsilon_{1}a_{2}\rho \right)\lambda_{2}}-\frac{y}{\lambda_{f_{2}}}\right)dy\\ =1-\sum_{k=1}^{K}\left(\begin{array}{c} K\\ k \end{array}\right){\left(-1\right)}^{k-1}\vartheta_{2}. \end{array}$$

It is noted that $\Xi_{3}$ can be formulated as

(A16) $$\begin{array}{c} \Xi_{3}=1-\mathrm{Pr}\left(\chi_{D2}^{PDMA}\geq \varepsilon_{1}\right)\\ =1-\mathrm{Pr}\left({\left|h_{2}\right|}^{2}\geq \frac{\varepsilon_{1}\varpi \rho {\left|f_{2}\right|}^{2}+\varepsilon_{1}}{\rho }\right)\\ =1-\frac{1}{\lambda_{f_{2}}}\int_{0}^{\infty }\exp\left(-\frac{\varepsilon_{1}\varpi \rho y+\varepsilon_{1}}{\rho \lambda_{h_{2}}}-\frac{y}{\lambda_{f_{2}}}\right)dy\\ =1-\frac{\rho \lambda_{h_{2}}}{\rho \lambda_{h_{2}}+\varepsilon_{1}\varpi \rho \lambda_{f_{2}}}\exp\left(-\frac{\varepsilon_{1}}{\rho \lambda_{h_{2}}}\right). \end{array}$$

This completes the proof of Lemma 2. □
